# Exploring the potential impact of adding upper limit single trigger MET thresholds to a paediatric early warning scoring tool at a tertiary children's hospital: a retrospective review

**DOI:** 10.3389/fped.2024.1378637

**Published:** 2024-07-05

**Authors:** Shawn Steckle, Casey Fowler, Victoria Campbell

**Affiliations:** ^1^ Intensive Care Unit, Sunshine Coast University Hospital, Birtinya, QLD, Australia; ^2^ Rapid Response System Coordination Unit, Queensland Children’s Hospital, South Brisbane, QLD, Australia

**Keywords:** MET, early warning score, early warning tool, CEWT, track and trigger system

## Abstract

**Objective:**

This study aims to determine the impact of incorporating upper threshold vital sign triggers into the digital Children's Early Warning Tool (CEWT) on the number of medical emergency team (MET) alerts.

**Methods:**

De-identified vital set data from the Cerner Millennium Integrated Electronic Medical Records were obtained for all paediatric patients aged ≤16 years at a tertiary children's hospital in Brisbane over a 12-month period in 2022. Patients in the paediatric intensive care unit, post-anaesthetic care unit, or the emergency department were excluded as they would not trigger MET alerts in these locations. Microsoft Excel scripts were used to tabulate and graph the data to compare the number of MET alerts in the current system vs. the system with proposed upper thresholds for heart rate, respiratory rate, systolic blood pressure, and severe respiratory distress.

**Results:**

A total of 389,352 vital sets were used for analysis after exclusions. Total cumulative MET alerts increased by 229% from 1,707 to 5,623. The number of increased alerts was inversely proportional to the age group. Respiratory rate and systolic blood pressure were the vital signs most associated with increased alerts. The largest number of new alerts came from patients with lower CEWT scores, while the largest proportional increase in alerts came from those with higher CEWT scores.

**Conclusions:**

Incorporating upper threshold vital sign triggers into the digital CEWT leads to a substantial increase in MET alerts. The consequent workload is not justified, given the lack of evidence suggesting a failure of the current CEWT system in recognising deteriorating patients.

## Introduction

Paediatric early warning tools (PEWTs) assist in recognising early deterioration by detecting vital sign derangement, with the severity determining to whom and how to escalate the patient's care. Many different PEWTs exist: single-parameter threshold triggers [such as Between the Flags (pBTF) in New South Wales and the Victorian Children's Tool for Observation and Response (VICTOR) in Victoria], cumulative parameter scores [such as British Paediatric Early Warning Score (PEWS)], and hybrid combinations of the two [such as Children's Early Warning Tool (CEWT) in Queensland, Australia]. Although validated internationally, there remains varying evidence regarding the impact of PEWTs on patient-centred outcomes such as in-hospital cardiac arrests, in-hospital mortality, admission numbers, duration in paediatric intensive care unit (PICU), and duration of hospital stay (1–11). The evidence is less clear on whether escalation is best achieved through cumulative scoring systems or those with absolute thresholds for some parameters (1–5, 10, 12, 13). Multiple considerations arise when contrasting these structures: the presence of a tiered ward-level response prior to medical emergency team (MET) alerts, the intrusiveness of MET alerts, the impact of the number of MET alerts on workload for MET team members, whether more MET alerts improve recognition of deterioration, and whether earlier recognition changes patient outcomes.

**Figure 1:**
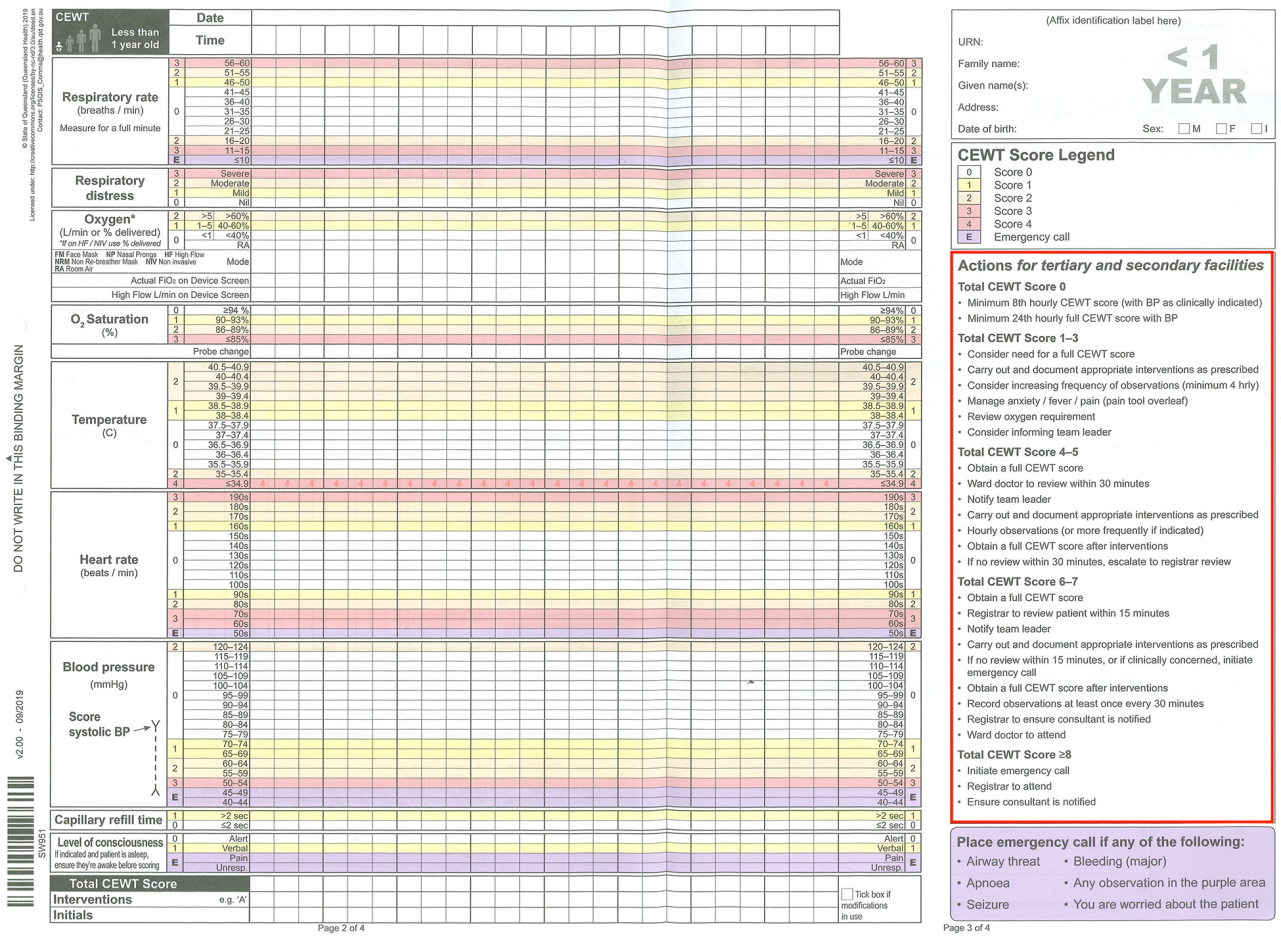
Paper CEWT score for age <1 year, used in Queensland for digital downtime or in any regional hospitals remaining in paper-based systems. The red box highlights the usual CEWT score-based actions that apply for both paper and digital CEWT. The purple zones denoted by “E” or the information in the purple text box are triggers for a MET response in the current system.

Queensland's CEWT is a hybrid multi-trigger tool with thresholds determined from local deterioration data. It was designed based on heuristic research with multiple studies demonstrating a favourable balance in recording and interpreting vital signs (5, 14). Its paper format was introduced state-wide by late 2010 across all public sites and has since been adopted by the private networks as well (8). Figure 1 shows an example of the CEWT chart for children aged <1 year.

The *digital* CEWT was introduced in 2016 with the introduction of the Cerner Millennium Integrated Electronic Medical Record (ieMR) and has been live in eight mixed-cohort hospitals and one tertiary children's hospital since 2018. The current digital CEWT includes a mixed system of age-specific cumulative scoring and lower-limit emergency thresholds for the respiratory rate (RR), heart rate (HR), and systolic blood pressure (SBP) based on the original paper charts. Every vital sign for every patient is entered into the ieMR, which uses a custom set of discern rules derived from the age-based CEWT to calculate an early warning score (EWS). Since CEWT is a multi-trigger tool, it generates an EWS and subsequent escalation alert for *either* cumulative mild to moderate vital sign derangements (like PEWS) *or* one or more severe vital sign derangements (like pBTF). Mild to moderate derangements generate an EWS of 1–7, requiring a ward-level response. Severe derangements of ≥1 vital sign(s) corresponding to “E” for emergency *or* an EWS ≥8 trigger a MET response.

The minimum set of vital signs required to generate an EWS in the digital CEWT are listed in Table 1. If fewer are entered, an “incomplete observations” alert prompts the clinician to enter a complete vital set to generate an EWS. For safety reasons, this “incomplete observations” alert is over-ridden by a MET alert if any single vital sign entered meets its lower threshold trigger (“E” purple zones in Figure 1). This occurs even if the triggering vital sign is the only one entered in the given vital set. There is currently no upper threshold trigger for any vital sign that has been a source of controversy—many sites across Australia use a single track and trigger PEWT (e.g., pBTF and VICTOR) and have been reluctant to change to a PEWT that is not inclusive of equivalent single trigger thresholds. Our study looks to further consider cumulative vs. threshold scoring regarding the effect on the number of MET alerts. We hypothesised that adding upper thresholds to RR, HR, SBP, and severe respiratory distress would substantially increase MET alerts.

**Table 1:** EWS vital set definitions with vital sign entry requirements to meet each definition, along with caveats to each.

	Vital signs entered	Caveats
Full EWS vital set	All nine vital signs: RR, respiratory distress, oxygen flow rate (or FiO_2_ if on high flow oxygen), oxygen saturation, temperature, HR, SBP, capillary refill, AVPU.	Must be entered in a single time column.
Partial EWS vital set	All six vital signs: RR, respiratory distress, oxygen flow rate (or FiO_2_ if on high flow oxygen), oxygen saturation, HR, capillary refill.	Intended for stable patients. Must be entered in a single time column Can exclude SBP, temperature, AVPU using clinical discretion. If this partial score is >0, the clinician is prompted to perform and enter a full EWS vital set.
Single vital sign MET trigger	Any single vital sign meeting MET trigger.	
Non-EWS vital set	If less than the partial EWS vital set is entered, e.g., one or two vital signs only.	Displays “incomplete observations” alert. No score will be generated unless a single vital sign among those entered meets a MET trigger for that vital sign.

AVPU, alert, verbal, pain, unresponsive scale.

## Methods

After attaining approval from the ethics committee and governance, vital sign data were collected for all paediatric patients at Queensland's largest tertiary paediatric hospital from 1 January 2022 to 31 December 2022. Patients in the emergency department (ED), operating theatre (OT), post-anaesthetic care unit (PACU), and PICU were excluded, as these locations are managed by internal emergency response processes rather than triggering a MET response. Children aged >16 years were excluded because Queensland uses its adult early warning tool instead of CEWT for these patients.

Every entered vital sign is stored in a Queensland Health data warehouse. Using a custom script in Microsoft SQL Server Management Studio v17 (Microsoft, Redmond, WA, USA), a data table was constructed containing all vital sets taken from the ieMR. The data table was then imported into QlikView 12 (Qlik, King of Prussia, PA, USA) for matching and alignment into discrete time columns to ensure each vital sign was aligned with others taken from the same patient at the same point in time. A “vital sign entry” was defined as any time column with at least one of HR, SBP, RR, and respiratory distress recorded because these were the vital signs to which the proposed upper threshold triggers were added. Time columns without any of these vital signs were thus excluded as they were unchanged. Vital sign entries were then categorised as scoring (full vital sets with EWS, partial vital sets with EWS, or single vital sign triggers) or non-scoring (non-EWS vital sets or single vital signs below triggers). Duplicate vital sets where multiple parameters of similar values are recorded simultaneously were excluded. From the data collection, there was no way to determine which vital sign entries had scoring modifications in place.

Once the data were aligned and filtered, it was completely de-identified and exported into Microsoft Excel (Microsoft, Redmond, WA, USA). A series of scripts were written in Excel to calculate the current EWS based on CEWT age-based thresholds, as exemplified in Figure 1. This generated a score for either a full EWS vital set, a partial EWS vital set, or a single low vital sign trigger per the current CEWT. A second set of scripts calculated what the EWS would be with the proposed upper threshold triggers, as outlined in Table 2. These values represent the parameters above those that currently score a 3 on the digital CEWT (thus being “off the chart”), with anything below these upper limits continuing to score as per the current standard (the top value on the chart, being either a 2 or 3). A third script compared the current CEWT and the proposed CEWT with upper threshold triggers for impact assessment. The outcomes were impact on MET alert frequency (representing alert/escalation burden) and non-EWS vital sign MET alerts (representing potentially unrecognised derangement). Due to the de-identified nature of the data, consequent direct patient-centred outcomes were outside the scope of this review.

**Table 2:** Vital sign parameters for upper limit MET alert thresholds.

Parameter	<1 year	1–4 years	5–11 years	12–16 years
Respiratory rate	>60	>55	>50	>45
Respiratory distress	Severe	Severe	Severe	Severe
Heart rate	>200	>180	>180	>170
Systolic blood pressure	>125	>130	>135	>155

QADDS, Queensland Adult Deterioration Detection Score, is the early warning score used for all patients age >16 years.

## Results

**Figure 2:**
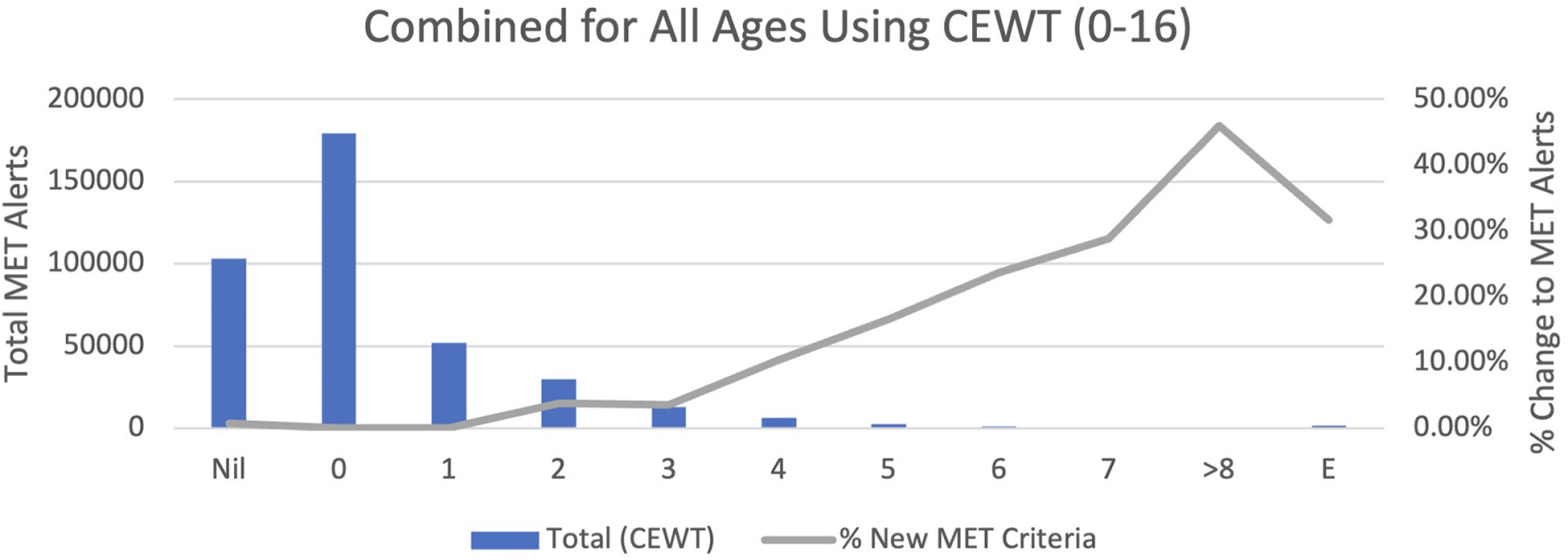
Total MET alerts and % change to MET alerts with upper limit thresholds vs. each EWS value from Nil to E, for all Children’s Early Warning Tool (CEWT data that include children aged 0–16.

In total, 389,677 vital sets were considered, with 325 duplicate entries excluded, leaving 389,352 vital sets. The results are combined in Table 3 and Figure 2, and then displayed by age group in Supplementary Material Tables 4–7 and Supplementary Material Figures 3–6. Across all age groups and CEWT scores combined, total MET alerts increased from 1,707 to 5,623, an increase of 3,916 alerts. The change of MET alerts per vital set of 0.44%–1.44% broadly represents a 229% increase. Considering the individual groups of age <1, 1–4, 5–11, and 12–16 years, MET alerts increased by 642%, 281%, 139%, and 51%, respectively.

**Table 3:** Data for all ages 0–16 years, representing all CEWT data—vital sign sets with both number and % difference of MET alerts, broken down by change per EWS and individual vital sign.

All ages 0–16 years combined			EWS	Total alerts	New alerts	% New MET criteria	EWS	RR	RD	HR	SBP
Vital sign sets	**3,89,677**		**Nil**	1,03,359	817	**0**.**79**	**Nil**	275	59	86	405
Remove dual entry	**3,89,352**	**325**	**0**	1,79,453	40	**0**.**02**	**0**	0	0	2	38
Current MET alerts	**1,707**	**0**.**44%**	**1**	51,954	9	**0**.**02**	**1**	3	0	1	5
Added proposed MET alerts	**3,916**	**1**.**01%**	**2**	29,917	1,152	**3**.**85**	**2**	1	0	2	1,150
New total	**5,623**	**1**.**44%**	**3**	12,733	455	**3**.**57**	**3**	117	5	49	284
			**4**	6,166	644	**10**.**44**	**4**	503	12	28	101
			**5**	2,612	433	**16**.**58**	**5**	311	14	41	71
			**6**	1,011	239	**23**.**64**	**6**	190	10	25	17
			**7**	440	127	**28**.**86**	**7**	87	7	28	9
			**>8**	254	117	**46**.**06**	**>8**	75	18	44	8
			**E**	1,453	460	**31**.**66**	**E**	225	15	197	38
			**Total**	**3,89,352**	**4,493**	**1**.**15**	**Total**	**1,787**	**140**	**503**	**2,126**
							**%**	**0.46%**	**0.04%**	**0.13%**	**0.55%**

RD, respiratory distress; % all VS, alerts as a percentage of total vital sets.

The distribution of MET alerts by current CEWT score is outlined in Table 3 and Supplementary Material Tables 4–7, and Figure 2 and Supplementary Material Figures 3–6. In total, there were 4,493 MET alerts due to the proposed thresholds. Of these, 577 correspond with CEWT scores ≥8 or E, so these are redundant alerts. Of the remaining 3,916, 1,443 alerts (37% of new MET alerts, 0.4% of all vital sets) had CEWT scores of 4–7, which would already trigger a ward-level review in the current system, and 1,616 alerts (41% of new MET alerts, 0.4% of all vital sets) had CEWT scores of 1–3, which already trigger nursing interventions and escalation to the nursing team leader in the current system, as described in Figure 1. Finally, 817 alerts (21% of new MET alerts, 0.2% of all vital sets) would not have received a CEWT score in the current system due to incomplete vital set entry.

The distribution of increased MET alerts by vital signs is also outlined in Table 3 and Supplementary Material Tables 4–7. By age, the youngest groups were the greatest contributors to new alerts, with new alerts inversely proportional to age. By vital signs, a high respiratory rate was responsible for most of the increased alerts in children aged <1 year. In the remaining age groups, both high respiratory rate and high systolic blood pressure caused the greatest alert increases. By EWS severity, MET alert numbers were inversely proportional to EWS (from pre-MET scores of 2–7).

## Discussion

This is the first review of its kind to consider the implications of adding upper threshold triggers to CEWT and suggests this to be a questionable use of health resources. MET alerts more than tripled with the proposed thresholds. In reviewing the available state-wide Severity Assessment Code (SAC) 1 reports from the Queensland Paediatric Quality Council (QPQC) from 2012 until 2019, there has never been a SAC 1 incident attributable to the inadequacy of the current CEWT thresholds (15, 16). Similar reports from 2019 to the present are not available at the time of writing. A SAC 1 is defined as death or likely permanent harm, which is not reasonably expected as an outcome of healthcare (17). Therefore, although the proposed upper thresholds substantially increased MET alerts as hypothesised, this increase and subsequent additional workload for the MET team cannot be justified based on the available evidence (1).

The digital CEWT follows the same score-based escalation criteria across age groups as the paper CEWT, as shown in Figure 1. For scores 1–3, an entire CEWT score must be calculated and escalation to the nursing team leader is prompted. For scores ≥4, a review by a doctor is required and an entire CEWT score must be calculated. Necessitating an entire vital set increases the likelihood of subsequently scoring higher to prompt even more rapid escalation. Of the new alerts, 78% were in patients with CEWT scores 2–7. There was a clear trend across age groups of MET alerts as a percentage of total vital sets increasing proportional to the CEWT score, particularly scores ≥4. These patients would thus receive prompt escalation in the current system, with deterioration already highly likely to be recognised. The highest volume of increased alerts occurred at CEWT scores 2–5, indicating much of the workload associated with the increased alerts would be for less sick patients who already receive appropriate escalation. None of the proposed upper thresholds make a new MET alert possible for CEWT scores 0–1. These new alerts most likely come from patients with CEWT score modifications applied by the treating doctor or “not for MET calls” limitations in place in their resuscitation plans. The applied changes subsequently alter the CEWT scoring from the baseline parameters in Table 2. Preventing this escalation is the point of those modifications; thus, this group accounting for 1% of the increased alerts could be excluded from analysis.

Finally, 817 patients with no CEWT score due to incomplete vital sets would now MET alert with the proposed thresholds. They account for the remaining 21% of new alerts and 0.2% of all vital signs. These are the patients whose deterioration the current system could theoretically miss and may stand to benefit from the proposed thresholds. However, the fact that no SAC 1 events have been attributed to the failure of the current thresholds would indicate that they are receiving appropriate escalation in the current system at some point in their trajectory. Future research using identifiable data is required to investigate their clinical outcomes and correlation with our alert-based findings.

The proposed thresholds increased MET alerts across all age groups, with the magnitude of increase inversely related to age. Younger patients were more likely to have non-EWS vital sets (see Table 1) entered. While RR was the vital sign most associated with new alerts, with the largest proportion in age <1 year, most of these new alerts were in non-EWS vital sets. This is important since tachypnoea in infants is non-specific and may represent respiratory, cardiovascular, metabolic, or systemic disease; this is why it is the most common single vital sign derangement in this cohort, serving as a compensatory mechanism (18, 19). It is difficult to explain why there were so many new alerts in this subset without cross-referencing patient charts, a process outside the scope of this review as it would require re-identifying data. It is recognised that the under 1-year age group is not homogenous, and these findings advocate for considering further age-defining RR thresholds in this age bracket.

SBP was the other vital sign most associated with new MET alerts across ages. Justification for these specific alerts is difficult to support. The definition of hypertension in children is not based on outcome measures as in adults but rather on the upper distribution of normal blood pressure (BP) from previous nomograms. Further evidence is required to determine accurate targets based on outcomes since increasing evidence suggests that paediatric BP depends on many contributing factors such as age, weight, gender, height, birth weight, and perinatal considerations (20). Assessment of BP alone also does not consider other vital parameters or markers of end-organ dysfunction that may indicate its significance. Extremes of BP often serve as late clinical markers in children due to their capacity to compensate and correlate less effectively than other signs with deterioration (13, 19–20). It may therefore be more efficient, as in the current system, for high SBP readings to prompt the need for complete vital set assessment to establish a CEWT score rather than triggering a MET alert in the absence of other data. Finally, measurement of BP is generally a specific challenge in many children. They often become distressed during the application and inflation of the cuff, which can lead to falsely elevated readings or abandonment of the attempt. This is another reason why BP is not a mandatory routine measurement in many PEWT systems (13).

### Strengths

CEWT is used in all Queensland digital sites, and the SAC 1 data previously mentioned included data from tertiary, regional, and rural sites. The data set comes from the largest paediatric centre in Queensland, making it the largest available to evaluate digital CEWT as a PEWT. While serious adverse events are a limited marker of potential harm, obtaining the state-wide SAC 1 data to draw inferences from the comparison is critical to internal and external validity.

### Limitations

Our data reflect only alerting thresholds and do not consider how this translates into patient care, actual MET call numbers, or clinical escalation. It is recognised that clinical escalation does not always occur as per protocol within the guidelines of early warning systems, and CEWT is no exception**.** Although there were no SAC 1 cases attributable to inadequacy of current CEWT thresholds, there were some sepsis cases identified as having issues in both staff recognition of deterioration using the CEWT and appropriate escalation based on its guidelines (14). Further analysis of the human factors contributing to this clinical practise issue was outside the scope of our review, but our findings encourage further research in this area.

The lack of identified data prevents us from linking new alerts with clinical incidents. This would be most useful for the 817 patients without a CEWT score in the current system who are most at risk of missed deterioration. Identified data would also be useful to confirm vital sets with score modifications in place and to further investigate the particular significance of the new alerts due to high RR in the <1 age group. It would be of particular interest to know what proportion of age <1 patients were admitted with bronchiolitis since tachypnoea can be out of proportion to other vital derangements whilst still using the same general CEWT thresholds defined in Table 2 and because they already trigger frequent escalation of care in their clinical trajectory.

Finally, this review is from a single tertiary site, which may limit the external validity of its conclusions, particularly to smaller centres with different staff resources and experience levels.

## Conclusions

Our review shows a substantial increase in MET alerts by incorporating upper limit thresholds into digital CEWT scoring. We believe the subsequent increased workload is unjustified, given the lack of reported events in paediatric SAC 1 cases attributable to inadequate current thresholds. Further research is required to identify the optimal PEWT and deterioration response systems and to investigate the consequences of direct patient-centred outcomes.

## Data Availability

The raw data supporting the conclusions of this article will be made available by the authors without undue reservation.
