# Supplementary material for: Exploring the potential impact of adding upper limit single trigger MET thresholds to a paediatric early warning scoring tool at a tertiary children's hospital: a retrospective review
Source: Front Pediatr. 2024 Jul 5;12:1378637. doi: 10.3389/fped.2024.1378637 (PMC11257883; doi:10.3389/fped.2024.1378637)
Supplement: Supplementary file 1 [file Datasheet1.pdf]

Table S1: Data for Age <1 – vital sign sets with both number and % difference of Medical Emergency Team (MET) alerts, broken down by change per Early Warning Score (EWS) and by individual vital sign. EWS = Early Warning Score. RR = Respiratory Rate. RD = Respiratory Distress. HR = Heart Rate. SBP = Systolic Blood Pressure. % all VS = alerts as a percentage of total vital sets.

Age <1		
Vital Sign Sets	67758	
Remove Dual Entry	67745	13
Current MET Alerts	226	0.33%
Added Proposed MET Alerts	1452	2.14%
New Total	1678	2.48%

EWS	Total Alerts	New Alerts	% New Criteria
Nil	15874	234	1.47%
0	27833	5	0.02%
1	11700	2	0.02%
2	6128	197	3.21%
3	2816	137	4.87%
4	1864	418	22.42%
5	796	234	29.40%
6	357	147	41.18%
7	151	78	51.66%
>8	78	56	71.79%
E	148	20	13.51%
Total	67745	1528	2.26%

EWS	RR	RD	HR	SBP
Nil	172	8	6	48
0	0	0	0	5
1	0	0	0	2
2	0	0	0	197
3	91	4	9	33
4	388	9	8	13
5	204	8	11	11
6	126	10	8	4
7	64	6	9	3
>8	37	15	22	4
E	16	2	1	0
Total	1098	62	74	320
% all VS	1.62%	0.09%	0.11%	0.47%

Table S2: Data for Age 1-4 – vital sign sets with both number and % difference of Medical Emergency Team (MET) alerts, broken down by change per Early Warning Score (EWS) and by individual vital sign. EWS = Early Warning Score. RR = Respiratory Rate. RD = Respiratory Distress. HR = Heart Rate. SBP = Systolic Blood Pressure. % all VS = alerts as a percentage of total vital sets.

Age 1-4		
Vital Sign Sets	114435	
Remove Dual Entry	**114352**	**83**
Current MET Alerts	**559**	**0.49%**
Added Proposed MET Alerts	**1572**	**1.37%**
New Total	**2131**	**1.86%**

EWS	Total Alerts	New Alerts	% New MET Criteria
**Nil**	31201	354	**1.13%**
**0**	47702	15	**0.03%**
**1**	15898	1	**0.01%**
**2**	10468	534	**5.10%**
**3**	4728	207	**4.38%**
**4**	2222	183	**8.24%**
**5**	1005	162	**16.12%**
**6**	394	77	**19.54%**
**7**	175	39	**22.29%**
**>8**	109	57	**52.29%**
**E**	450	127	**28.22%**
**Total**	**114352**	**1756**	**1.54%**

EWS	RR	RD	HR	SBP
**Nil**	73	11	65	208
**0**	0	0	0	15
**1**	1	0	0	0
**2**	0	0	2	533
**3**	20	0	33	154
**4**	111	2	18	52
**5**	100	5	25	33
**6**	52	0	13	12
**7**	20	0	16	5
**>8**	36	3	20	4
**E**	31	9	80	20
**Total**	**444**	**30**	**272**	**1036**
**%**	**0.39%**	**0.03%**	**0.24%**	**0.91%**

Table S3: Data for Age 5-11 – vital sign sets with both number and % difference of Medical Emergency Team (MET) alerts, broken down by change per Early Warning Score (EWS) and by individual vital sign. EWS = Early Warning Score. RR = Respiratory Rate. RD = Respiratory Distress. HR = Heart Rate. SBP = Systolic Blood Pressure. % all VS = alerts as a percentage of total vital sets.

Age 5-11		
Vital Sign Sets	113158	
Remove Dual Entry	**113089**	**69**
Current MET Alerts	**478**	**0.42%**
Added Proposed MET Alerts	**666**	**0.59%**
New Total	**1144**	**1.01%**

EWS	Total Alerts	New Alerts	% New MET Criteria
**Nil**	28732	134	**0.47%**
**0**	54563	13	**0.02%**
**1**	15399	4	**0.03%**
**2**	8641	339	**3.92%**
**3**	3317	89	**2.68%**
**4**	1289	37	**2.87%**
**5**	484	31	**6.40%**
**6**	142	13	**9.15%**
**7**	44	6	**13.64%**
**>8**	15	4	**26.67%**
**E**	463	185	**39.96%**
**Total**	**113089**	**855**	**0.76%**

EWS	RR	RD	HR	SBP
**Nil**	14	2	8	110
**0**	0	0	1	12
**1**	1	0	1	2
**2**	1	0	0	338
**3**	1	1	3	84
**4**	2	0	1	34
**5**	7	0	2	23
**6**	10	0	2	1
**7**	2	0	3	1
**>8**	2	0	2	0
**E**	82	2	100	3
**Total**	**122**	**5**	**123**	**608**
**%**	**0.11%**	**0.00%**	**0.11%**	**0.54%**

Table S4: Data for Age 12-16 – vital sign sets with both number and % difference of Medical Emergency Team (MET) alerts, broken down by change per Early Warning Score (EWS) and by individual vital sign. EWS = Early Warning Score. RR = Respiratory Rate. RD = Respiratory Distress. HR = Heart Rate. SBP = Systolic Blood Pressure. % all VS = alerts as a percentage of total vital sets.

Age 12-16		
Vital Sign Sets	94326	
Remove Dual Entry	**94166**	**160**
Current MET Alerts	**444**	**0.47%**
Added Proposed MET Alerts	**226**	**0.24%**
New Total	**670**	**0.71%**

EWS	Total Alerts	New Alerts	% New MET Criteria
**Nil**	27552	95	**0.34%**
**0**	49355	7	**0.01%**
**1**	8957	2	**0.02%**
**2**	4680	82	**1.75%**
**3**	1872	22	**1.18%**
**4**	791	6	**0.76%**
**5**	327	6	**1.83%**
**6**	118	2	**1.69%**
**7**	70	4	**5.71%**
**>8**	52	0	**0.00%**
**E**	392	128	**32.65%**
**Total**	**94166**	**354**	**0.38%**

EWS	RR	RD	HR	SBP
**Nil**	16	38	7	39
**0**	0	0	1	6
**1**	1	0	0	1
**2**	0	0	0	82
**3**	5	0	4	13
**4**	2	1	1	2
**5**	0	1	3	4
**6**	2	0	2	0
**7**	1	1	0	0
**>8**	0	0	0	0
**E**	96	2	16	15
**Total**	**123**	**43**	**34**	**162**
**%**	**0.13%**	**0.05%**	**0.04%**	**0.17%**

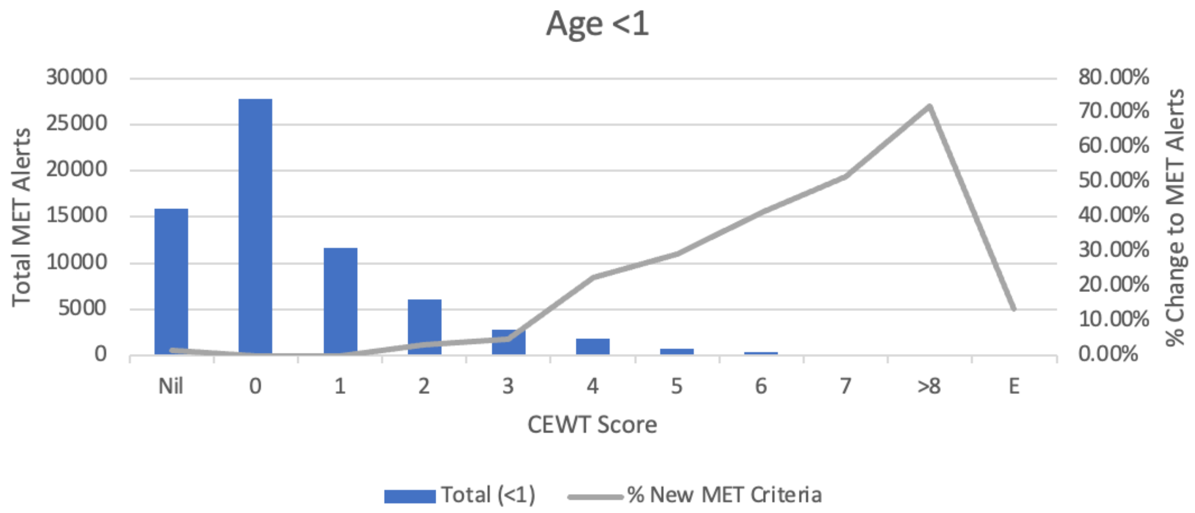

Figure S1: Total Medical Emergency Team (MET) alerts and % change to MET alerts with upper limit thresholds vs each Early Warning Score (EWS) value from Nil – E, for Age <1.

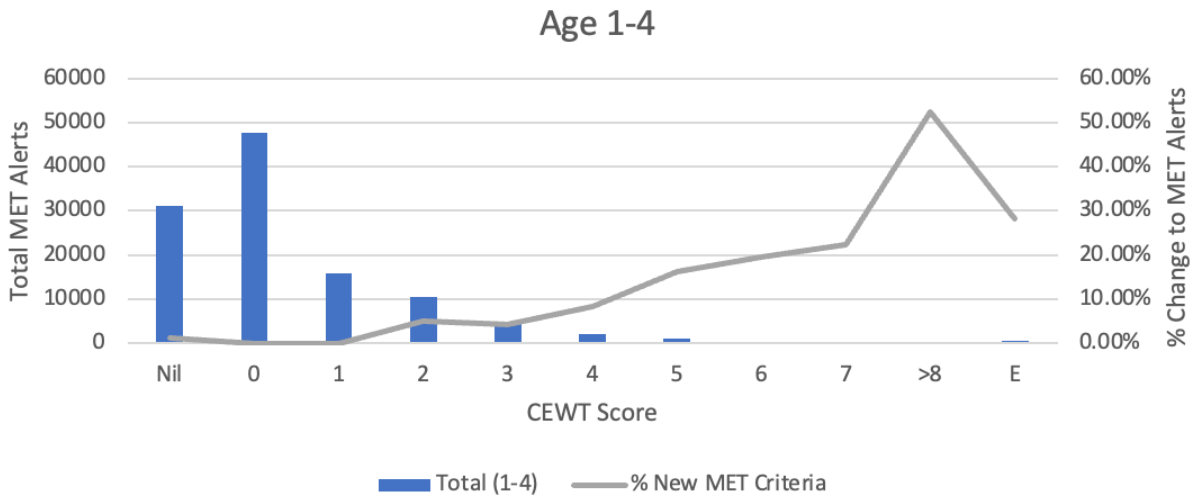

Figure S2: Total Medical Emergency Team (MET) alerts and % change to MET alerts with upper limit thresholds vs each Early Warning Score (EWS) value from Nil – E, for Age 1-4.

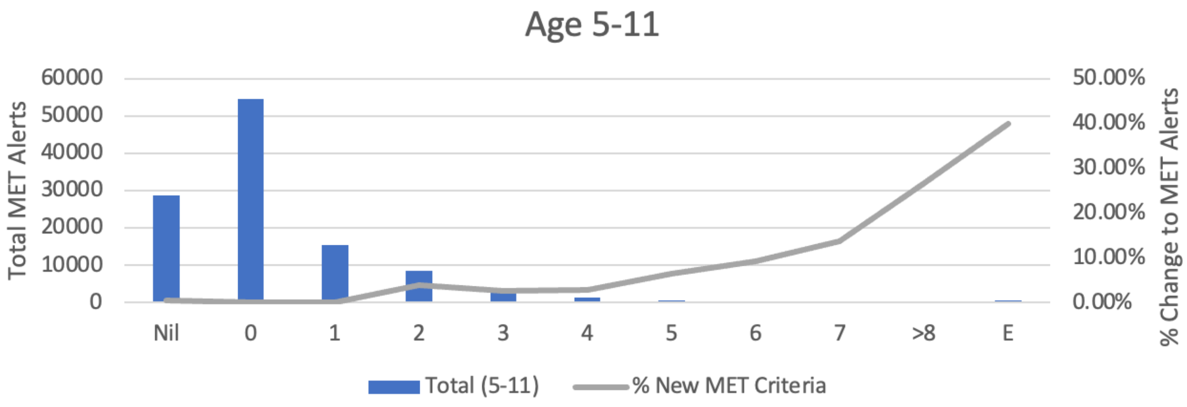

Figure S3: Total Medical Emergency Team (MET) alerts and % change to MET alerts with upper limit thresholds vs each Early Warning Score (EWS) value from Nil – E, for Age 5-11.

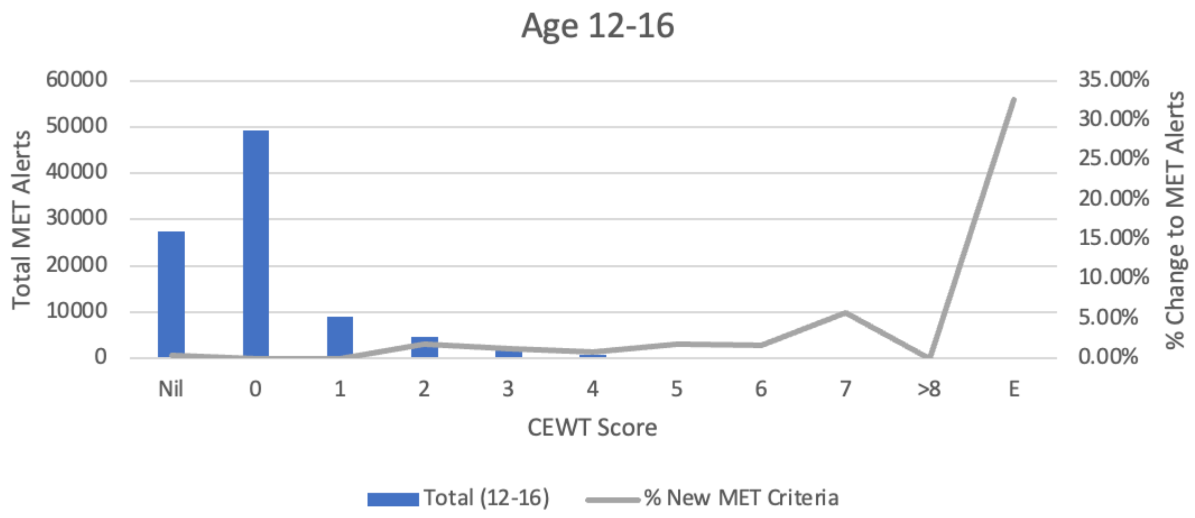

Figure S4: Total Medical Emergency Team (MET) alerts and % change to MET alerts with upper limit thresholds vs each Early Warning Score (EWS) value from Nil – E, for Age 12-16.
